# Case Report: Ischemic Colitis in Severe COVID-19 Pneumonia: An Unforeseen Gastrointestinal Complication

**DOI:** 10.4269/ajtmh.20-1262

**Published:** 2020-11-03

**Authors:** Theresa Paul, Antony Raphel Joy, Hussam Abdel Rahman S. Alsoub, Jessiya Veliyankodan Parambil

**Affiliations:** 1 Department of Internal Medicine, Hamad Medical Corporation, Doha, Qatar;; 2 Department of Gastroenterology, Hamad Medical Corporation, Doha, Qatar;; 3 Department of Infectious Diseases, Hamad Medical Corporation, Doha, Qatar

## Abstract

Clinical manifestations and complications of SARS-CoV-2 are still emerging and variant. Gastrointestinal (GI) manifestations and complications are hugely under-recognized. The presence of angiotensin converting enzyme-2 receptors in the intestinal enterocytes, the receptors primarily involved in the pathogenesis of COVID-19 pneumonia, maybe the key factor contributing to the pathogenesis of GI manifestations. Ischemic colitis, although the most common ischemic pathology of the GI tract, is relatively rare, occurring as a result of colonic hypoperfusion. The innumerable causes of colonic ischemia are categorized into occlusive and nonocclusive pathologies. Here, we have discussed a case of severe COVID-19 pneumonia, developing ischemic colitis, as a rare GI complication. The cause of ischemia in COVID-19 pneumonia is multifactorial, including hypercoagulable state, coagulopathy leading to thromboembolic complications, and use of vasopressors in severely ill patients with hemodynamic compromise.

## INTRODUCTION

COVID-19 was declared as a global pandemic by the WHO on March 11, 2020.^1^ Little was known about the virus, its pathogenesis, and its manifestations initially. Over the past few months, medical practitioners worldwide had been analyzing, studying, understanding, and contributing to the literature regarding the various manifestations and the unexpected and unforeseen complications of the disease. Even though the classical presentations are with upper and lower respiratory tract symptoms,^2^ numerous atypical presentations of the disease have been noticed over time. Of these, the gastrointestinal (GI) manifestations of the disease are increasingly being recognized. Some studies report around 50% of SARS-CoV-2 patients presenting with GI manifestations.^3^ The most common GI presentation in patients with COVID-19 was diarrhea, followed by nausea, vomiting, and abdominal pain. Others include anorexia, anosmia, and dysgeusia.^4^ Angiotensin converting enzyme-2 (ACE 2) receptors are the target viral receptors for SARS-CoV-2 in the pathogenesis of COVID-19 pneumonia, which are present abundantly in the alveolar cells. Their presence in the glandular cells of the gastric, duodenal, and rectal epithelium may be the critical factor contributing to the development of GI manifestations in COVID-19 pneumonia.^5^

Here, we present the case of a patient developing ischemic colitis as a rare and unforeseen GI complication in severe COVID-19 pneumonia.

## CASE REPORT

### Case 1.

A 66-year-old male patient, previously healthy, presented to the emergency department with a history of fever, cough, and loss of smell and taste for 3 days. A review of systems was unremarkable. Examination showed a well-built male, tachypneic with a respiratory rate of 32 breaths/minute. He was afebrile, heart rate was 90 beats/minute, and blood pressure 108/64 mmHg. Respiratory system examination revealed hyperemic pharynx and bilateral basal crackles on auscultation. The patient deteriorated rapidly in the emergency room, developing severe hypoxic respiratory failure leading to intubation and mechanical ventilation. The initial laboratory results had normal blood cell counts, normal liver and renal function, and high ferritin of 1,086 mcg/L.

Chest X-ray performed showed bilateral pneumonic patches and basal atelectasis. Reverse transcriptase–PCR from nasopharyngeal swab for SARS-CoV-2 was positive.

He was admitted to the medical intensive care unit (ICU) as a case of severe COVID-19 pneumonia and started on azithromycin 500 mg, cefuroxime 1.5 g q8 hourly, hydroxychloroquine 400 mg, and methylprednisolone 40 MG twice a day, which was the local treatment protocol for COVID-19 pneumonia at that time. He also received tocilizumab and convalescent plasma for severe Acute Respiratory Distress Syndrome because of COVID-19 pneumonia on day 5 of admission. On day 8 of hospital stay, the patient deteriorated, and developed hypotension and diarrhea. Vasopressor support with noradrenaline was initiated, and antibiotics were escalated to piperacillin–tazobactam and teicoplanin along with anidulafungin for antifungal coverage. Urine culture grew *Pseudomonas aeruginosa* and *candida*, and sputum culture grew methicillin-resistant *Staphylococcus aureus* and *Klebsiella pneumoniae*. Vasopressor was titrated down and discontinued on day 4 after initiation, as the patient started to improve. He was extubated the next day successfully and transferred to the high-dependency unit for further care. Five days after de-escalation of care, the patient developed melena and severe fresh bleeding per rectum with passage of clots. His laboratory investigations showed a rapid drop in hemoglobin to 4.7g/dL. Packed red blood cells were transfused, and gastroenterologist consulted. Urgent bedside endoscopic evaluation was performed. Esophagus to the second part of the duodenum showed normal mucosa with no altered blood. During sigmoidoscopy, scope was passed into the rectum, and fresh blood with clots was noted. Further passage of the scope to the sigmoid colon showed large clots with multiple ulcerations, probably ischemic, and biopsies were taken. No further overt bleed noted after washing, however, could have similar changes higher up.

Gastroenterologist recommended conservative supportive management with blood transfusion as no intervention was possible. He had further melena 5 days later, which stabilized with conservative measures. Since then, the patient improved well; no further bleeding was observed; he stayed hemodynamically stable and was discharged home on day 40 of admission.

## DISCUSSION

Ischemic colitis, although the most common ischemic pathology of the GI tract, is relatively rare, occurring as a result of colonic hypoperfusion.^6,7^ When there is insufficient blood to maintain cellular metabolic function, cellular destruction ensues, with colonocytes becoming acidotic, dysfunctional, and ultimately leading to cell death.

Ischemia of the colon can lead to reversible or irreversible damage, the former of which includes colopathy and colitis. Colitis occurs within 3 days when the overlying mucosal edema from colopathy is resorbed, resulting in mucosal ulceration, which takes several months to resolve, despite the patient being asymptomatic.^8^

The diagnosis of colon ischemia should be considered, and early colonoscopy is advised in patients presenting with severe abdominal pain followed by lower GI bleed in the next 12–24 hours.^9^ Some common but less specific symptoms include painless diarrhea, nausea, or vomiting. On physical examination, varying degree of tenderness is elicited over the affected region, depending on the extent of colonocyte damage; even signs of peritonitis, Systemic Inflammatory Response Syndrome (sepsis, tachypnea, and tachycardia) may be evident, along with augmenting laboratory findings (leukocytosis, metabolic acidosis, elevation of lactate, inorganic phosphate, and alkaline phosphate).^9–15^

The innumerable causes of colonic ischemia can be categorized into occlusive and nonocclusive pathologies. Thrombophilia and vasculitis are the most important among the occlusive causes of ischemic colitis in the young.^16^

SARS-CoV-2 has been linked to the development of coagulopathy and thromboembolic complications in severe COVID-19 patients. The presentations included pulmonary embolism, deep venous thrombosis, and rarely mesenteric ischemia. Deep vein thrombosis and pulmonary embolism have typical presentations and anticipated complications in severely ill patients, especially in ICU settings. However, the diagnosis of colonic ischemia remains a challenge with severe COVID-19 patients in ICU care, as the hallmark symptom of abdominal pain is masked and not easily recognized in intubated, sedated patients.^17^ Imaging findings in severe COVID-19 demonstrate bowel necrosis secondary to small vessel thrombosis, highlighting the fact that ischemia is often due to in situ thrombosis, rather than due to thromboembolic events.^18,19^ In situ thrombosis can also be attributed to the fact that there is an increase in the levels of von-Willebrand factor in severe COVID-19, leading to endothelial damage, activation, and thrombosis. In addition, the novel corona virus’s target viral receptor is the ACE-2, which is expressed abundantly not only in the alveolar cells, but also in the GI tract, which can explain the endothelial tropism and the resulting endothelial dysfunction or damage and thrombosis.^5,20^

Severe COVID-19 can lead to nonocclusive colonic ischemia, as shock and hemodynamic compromise are common in COVID-19 pneumonia. In patients requiring ionotropic support, there is intense activation of vasoconstrictor fibers through alpha-adrenergic stimulation resulting in vasoconstriction of small vessels and decreased mesenteric blood flow, further compromising colon tissue perfusion.^21^ Norepinephrine and high levels of epinephrine produce intense vasoconstriction through the stimulation of adrenergic receptors. Other pharmacologic compounds that decrease splanchnic blood flow include vasopressin, phenylephrine, and digoxin. However, it is not always vasoconstriction from ionotropic support; low doses of dopamine (3–8 μg/kg/minute) and epinephrine (0.05–0.10 μg/kg/minute) have been shown to increase the mesenteric blood flow and provide adequate vasopressor support, being protective to the bowel.^22^

The vibrant clinical presentation and complications of COVID-19 are varied and still emerging. In this case, the rare GI complication of ischemic colitis and its possible causes in COVID-19 are described. Severe COVID-19 pneumonia should be considered a hypercoagulable state. In critically ill patients, it is ideal to use vasopressors with caution, keeping in mind the ischemic complications, and diagnosing and managing them early in its course for better patient outcomes. This patient scenario again affirmed the need for further reports to understand and manage the under-recognized and unforeseen complications of SARS-CoV-2.
